# Integrating Cognitive Behavioral Group Therapy and Psychodrama for Social Anxiety Disorder: An Intervention Description and an Uncontrolled Pilot Trial

**DOI:** 10.32872/cpe.v2i1.2693

**Published:** 2020-03-31

**Authors:** Hanieh Abeditehrani, Corine Dijk, Mahdi Sahragard Toghchi, Arnoud Arntz

**Affiliations:** a Department of Clinical Psychology, University of Amsterdam, Amsterdam, The Netherlands; b Department of Psychology, Payame Noor University, Tehran, Iran; Philipps-University of Marburg, Marburg, Germany

**Keywords:** integrating therapies, cognitive behavioral therapy, psychodrama, social anxiety disorder, clinical trial

## Abstract

**Background:**

Cognitive behavioral therapy (CBT) is generally considered to be the most effective psychological treatment for social anxiety disorder (SAD). Nevertheless, many patients with SAD are still symptomatic after treatment. The present pilot study aimed to examine integrating CBT, with a focus on cognitive and behavioral techniques, and psychodrama, which focuses more on experiential techniques into a combined treatment (CBPT) for social anxious patients in a group format. This new intervention for SAD is described session-by-session.

**Method:**

Five adult female patients diagnosed with social anxiety disorder participated in a twelve-session CBPT in a group format. Pretest and posttest scores of social anxiety, avoidance, spontaneity, cost and probability estimates of negative social events, depression, and quality of life were compared, as were weekly assessments of fear of negative evaluation.

**Results:**

Results demonstrated a significant reduction of the fear of negative evaluation and social anxiety symptoms. It is noteworthy that also the scores of the probability and cost estimates decreased. However, there were no significant differences between pre and post measures in any of other measures.

**Conclusion:**

The current study suggests that group CBPT might be an effective treatment for SAD. However, our sample size was small and this was an uncontrolled study. Therefore, it is necessary to test this intervention in a randomized controlled trial with follow-up assessments.

## Abstract (highlights)

- This study describes integrating cognitive behavioral therapy and psychodrama (CBPT).
- CBPT significantly reduced fear of negative evaluation and social anxiety.
- Three of the five patients have a clinically significant change on the LSAS after the treatment.
- CBPT also changed estimates of social cost and the probability of negative social events.

Social anxiety disorder (SAD) is one of the most common mental disorders, with a 13% lifetime prevalence (Kessler, Petukhova, Sampson, Zaslavsky, & Wittchen, 2012). Recent research shows that the prevalence of SAD in Iran is approximately 10% (Talepasand & Nokani, 2010). Depression is highly comorbid with SAD and more than half of the patients report lifetime major depression (Brown, Campbell, Lehman, Grisham, & Mancill, 2001). SAD is associated with increased functional disability, substantial economic inactivity, and a lower quality of life (Patel, Knapp, Henderson, & Baldwin, 2002). Therefore, it is important to treat SAD effectively.

Several meta-analyses show that cognitive behavioral therapy (CBT) is the most effective psychotherapy for SAD (Hofmann & Smits, 2008; Mayo-Wilson et al., 2014). CBT is an eclectic approach based on a combination of techniques from cognitive and behavioral theories (Harwood, Beutler, & Charvat, 2001). Cognitive behavioral group therapy (CBGT), as developed by Heimberg and Becker (1991, 2002) is an efficacious and evidence-based treatment for SAD. The effect of CBGT on social anxiety symptoms has been demonstrated in meta-analyses (Barkowski et al., 2016; Mayo-Wilson et al., 2014). CBGT usually consists of cognitive restructuring, exposure and homework assignments (Coles, Hart, & Heimberg, 2005; Heimberg & Becker, 2002). Judgmental biases such as beliefs about the cost and probability of negative social events play an important role in the maintenance of SAD (Clark & Wells, 1995; Heimberg, Brozovich, & Rapee, 2010; Hofmann, 2007; Morrison & Heimberg, 2013). There is an association between CBT treatment and a reduction in probability or cost estimates for individuals with SAD (Foa, Franklin, Perry, & Herbert, 1996; Gregory, Peters, Abbott, Gaston, & Rapee, 2015; Hofmann, 2004; Lucock & Salkovskis, 1988; Poulton & Andrews, 1994). Hence, CB(G)T is an effective treatment for SAD. However, 25-50% of patients with SAD show little or no improvement after treatment (Davidson et al., 2004; Heimberg et al., 1998; Hofmann & Bögels, 2006). Thus, many patients remain symptomatic after completing treatment, and it is clear that there is room to improve interventions to enhance clinical outcomes for SAD.

We propose that CBT and psychodrama can be integrated to enhance treatment effects. Psychodrama is an action-based method of group psychotherapy, developed by Jacob Levy Moreno (Moreno, 1946). In psychodrama, patients use role-playing to dramatize their psychological and social problems rather than just talking about them (Blatner, 2000). Furthermore, psychodrama can enhance the potency of therapeutic alliance and create a therapeutic bond between group members by letting patients engage in role-playing and the playing of auxiliaries in the other members’ enactment, and by evoking emotions during action (Orkibi, Azoulay, Regev, & Snir, 2017). Several studies with non-SAD samples on the combination of CBT and psychodrama demonstrated that CBT and psychodrama could be integrated (Boury, Treadwell, & Kumar, 2001; Hamamci, 2002, 2006; Treadwell, Kumar, & Wright, 2002). There are several reasons why psychodrama techniques can enhance therapy outcome for SAD patients as well. First, several acting techniques in psychodrama do not occur in CBGT but might be helpful, because they involve experiential learning (see Table 2 for a description of typical psychodrama techniques and their goals for treatment of SAD), whereas the focus of traditional CBT is on cognitive and behavioral learning. Second, there is increasing evidence that (traumatic) childhood experiences contribute to the development of SAD (Arrindell, Emmelkamp, Monsma, & Brilman, 1983; Blöte, Miers, & Westenberg, 2015; Bruch & Heimberg, 1994; Kuo, Goldin, Werner, Heimberg, & Gross, 2011; Simon et al., 2009). Psychodrama provides an opportunity to reenact a negative social interaction from the past as if it occurs in the present, but now in the safe setting in which the patient has more control over what is said and done. This might, in turn, change the patient's beliefs, feelings, and attitudes about the traumatized situation (Treadwell & Kumar, 2002). Third, socially anxious people devote effort to control the expression of feelings and suppress their emotions to minimize the chance of making social transgressions and elicit rejection by others (Kashdan & Steger, 2006). They also report a fear of experiencing emotions and more negative beliefs about the consequences of emotional expression (Spokas, Luterek, & Heimberg, 2009). In psychodrama, a safe environment is created which can help patients to express their inhibited emotions and examine the accuracy of their beliefs about the negative outcomes of this. Finally, according to Moreno’s theory, anxiety decreases by increasing spontaneity. In CBT-terms, spontaneity can be seen as the opposite of avoidance and inhibition that is central to SAD. One of the aims of psychodrama is to increase spontaneity.

There is no research to demonstrate that CBT and psychodrama can be integrated into the treatment of social anxiety disorder. The main aim of this pilot study is to describe the intervention and examine the integrated group CBT-psychodrama protocol (labelled CBPT) to treat social anxious patients and to get a first impression of its effectiveness. We hypothesized that CBT and psychodrama can be successfully integrated and that this integration is effective in improving fear of negative evaluation, the characteristic feature of SAD, which was measured by the Brief Fear of Negative Evaluation Scale (BFNE), and social anxiety symptoms, which were measured by the Liebowitz Social Anxiety Scale (LSAS). Furthermore, integrating psychodrama and CBGT might be efficacious for SAD because they focus on separate mechanisms. Psychodrama focuses on increasing spontaneity and decreasing avoidance behavior through role-playing. CBGT, on the other hand, focuses on decreasing cognitive biases associated with SAD and decreases avoidance behavior through exposure. The CBPT, therefore, might offer a broader treatment which might also affect depression, an often comorbid disorder with SAD, and increase the quality of life in patients suffering from SAD.

## Method

### Participants

Six patients with a primary diagnosis of social anxiety disorder were included in this study; all were diagnosed with the Structured Clinical Interview for DSM 4th ed (SCID-I, Farsi Version; First, Spitzer, Gibbon, & Williams, 2012). Participants were recruited through the media and poster advertisements. One participant dropped out of the study because she found a full-time job before the first session, and was therefore not included in the analysis. All the patients were females, living in Tehran. The mean age of the five patients was 36.6 (age range = 21-63; *SD* = 17.89). Three of them were diagnosed with generalized and two of them with specific SAD. An Iranian ethical committee (reference number IR.UMSHA.REC.1394.521) approved the protocol on February 27, 2016, and all patients gave their written informed consent prior to their inclusion in the study. This study is a preparatory pilot for an RCT that included the CBPT protocol as an arm. The RCT was preregistered at a trial register (IRCT2016032321385N1). Inclusion criteria were SAD as a primary diagnosis, age between 18 and 65 years, ability to read and understand the questionnaires and the interview. Exclusion criteria were comorbid psychotic or bipolar disorder, lifetime history of schizophrenia or bipolar disorder, a high suicidality risk, antisocial or borderline personality disorder, a comorbid diagnosis of substance abuse or dependence. Furthermore, unwillingness to stabilize medication for the duration of the study was an exclusion criterion as well.

### Procedures and Measures

Social anxiety was assessed with the clinician-administered version of the LSAS (Liebowitz, 1987) at pre and posttests by an independent assessor and the Brief Fear of Negative Evaluation Scale (BFNE; Rodebaugh et al., 2004; Weeks et al., 2005) was completed before the treatment and also after every treatment session (thus in total there were 13 measurements). Additionally, the patients were assessed at pre and posttests on the following outcomes: social avoidance with the Social Avoidance and Distress Scale (SADS; Watson & Friend, 1969); spontaneity with the Personal Attitude Scale-II (PAS; Kellar, Treadwell, Kumar, & Leach, 2002); and cost and probability estimates of negative social events with the Outcome Probability Questionnaire (OPQ; Uren, Szabó, & Lovibond, 2004) and the Outcome Cost Questionnaire (OCQ; Uren et al., 2004). Depression was measured with the Beck Depression Inventory (BDI; Beck, Steer, & Brown, 1996), and quality of life was measured with the Quality of Life Inventory (QOLI; Frisch, Cornell, Villanueva, & Retzlaff, 1992).

For the several questionnaires, no Persian version existed (e.g., Quality of Life Inventory, Outcome Probability Questionnaire, Outcome Cost Questionnaire, and Personal Attitude Scale-II). Therefore, these were translated and back-translated to ensure the adequacy of the translation.

Finally, therapists used a session report form to record the procedures used in the session, such as the name of the protagonist and the auxiliaries, the type of therapeutic techniques that were used (e.g., role reversal, cognitive challenging), and also patients’ feedback on the therapy session.

#### Primary Outcomes

The Brief Fear of Negative Evaluation Scale (BFNE; Rodebaugh et al., 2004; Weeks et al., 2005), is a self-report measure consisting of 12 items on a 5-point Likert scale (1 = *strongly disagree*, 5 = *strongly agree*). An example question is: “I am afraid that others will not approve of me”. The BFNE has excellent internal consistency (Cronbach's alpha > .92) and validity in clinical samples (Weeks et al., 2005).

The Liebowitz Social Anxiety Scale – clinician-administered version (LSAS; Liebowitz, 1987) is a 24-item interview that assesses fear and avoidance, in social interactions (e.g., talking with people you don’t know very well) and performance situations (e.g., returning goods to a store). The items are on a 4-point-Likert scale (0 = *never*, 3 = *usually*). The LSAS has shown good test–retest reliability, internal consistency, and convergent and discriminant validity (Baker, Heinrichs, Kim, & Hofmann, 2002; Fresco et al., 2001; Oakman, Van Ameringen, Mancini, & Farvolden, 2003; Rytwinski et al., 2009).

#### Secondary Outcomes

The Social Avoidance and Distress Scale (SADS; Watson & Friend, 1969) is a self-report inventory with 28-item that includes 14 items to assess social avoidance (e.g., I often want to get away from people) and 14 items to assess social anxiety (e.g., I often feel on edge when I am with a group of people). All items are rated as true or false. Cronbach's alpha reliability coefficient was .90 and the test-retest reliability was .77 in a study by Watson and Friend (1969).

The Personal Attitude Scale-II (PAS; Kellar, Treadwell, Kumar, & Leach, 2002) is a self-report measure of spontaneity. An example item is: “I am at ease when meeting new people”. It has 66 items on a 5-point Likert scale (0 = *strongly disagree*; 4 = *strongly agree*). Cronbach's alpha reliability coefficient of internal consistency was .92 and the test-retest reliability was .86 in a study by Kellar et al. (2002).

The Outcome Probability Questionnaire (OPQ) and the Outcome Cost Questionnaire (OCQ) (Uren, Szabó, & Lovibond, 2004) are two self-report questionnaires consisting of 12 items. The OPQ assesses an individual’s probability estimate of the occurrence of negative social events (e.g., how likely would be for you at a party, others will notice that you are nervous?). The OCQ then asks about the same negative social events but here individuals are asked to indicate how costly it would be if these events were actually to occur (e.g., how distressing would be for you if at a party, others will notice that you are nervous?). Both questionnaires have items on a 9-point Likert scale (0 = *not at all likely/distressing*; 8 = *extremely likely/distressing*). The internal consistency of both instruments is good (Cronbach’s alpha ≥ .90) (Uren et al., 2004).

The Beck Depression Inventory-II (BDI-II; Beck, Steer, & Brown, 1996) is a 21-item self-report inventory that measures the severity of symptoms of depression in the previous two weeks (e.g., loss of energy, worthlessness). A good internal consistency (Cronbach’s alpha = .92), and test-retest reliability have been shown in several studies (Beck, Steer, & Carbin, 1988; Beck et al., 1996).

The Quality of Life Inventory (QOLI; Frisch, Cornell, Villanueva, & Retzlaff, 1992) is a 16-item self-report questionnaire that includes 16 areas that are related to the overall happiness of life (e.g., work, health). The survey asks the participants to describe first the importance (0 *= not at all important*, 2 = *very important*) and then satisfaction (+3 = *very satisfied*, -3 = *very dissatisfied*) of each area. For each area quality of life is measured by multiplying the importance with the satisfaction which can range from -6 to +6. The internal consistency is high, Cronbach’s alpha between α = 0.77 and α = 0.89, and the one month test-retest reliability is between *r* = 0.80 and *r* = 0.91 (Frisch et al., 1992).

#### Intervention

The CBPT therapists integrated cognitive restructuring and exposure with psychodrama techniques. The CBPT group underwent 12 weekly sessions each lasting 2.5 hours with five patients and two therapists (one male and one female). The therapists received training in the integrated psychodrama and CBT protocol, were trained in and had experience with conducting both psychodrama and CBGT. Furthermore, an expert in CBPT had weekly supervision meetings with the therapists to ensure the quality of the treatment. The CBPT treatment consisted of four phases: (1) an initial preparatory interview (2) building group cohesion and introduction of cognitive restructuring (Sessions 1 and 2), (3) CBT and psychodrama (Sessions 3 through 11), and (4) conclusion (the 12th session).

The treatment starts with an individual treatment orientation interview in which group treatment procedures and fear of participation in group sessions are discussed. This interview prepares patients for group sessions and makes them familiar with one of the therapists (Heimberg & Becker, 2002). Session 1 and 2 are devoted to creating a safe atmosphere in which patients can share their feelings and thoughts with other members of a group, and to the building of group cohesiveness. The sessions are based on Heimberg and Becker's (2002) CBGT protocol and are used as basic training in cognitive restructuring. In the first session, the therapists present CBPT therapy for social anxiety and briefly explain the primary treatment techniques. Next, the session focuses on the identification of automatic thoughts. At the end of the session, patients share their individual problems, and goals and homework are assigned, which is a recording of automatic thoughts during the following week. The second session is devoted to developing cognitive restructuring skills of patients and to introduce thinking errors by practicing with the recorded automatic thoughts form. The therapists teach patients how to dispute cognitions and replace negative automatic thoughts with more helpful cognitions. Therapists also inform and prepare patients for initiation of the role-playing in the third session. At the end of the session, homework is assigned again, which is to label thinking errors in the identified automatic thoughts and to practice with cognitive restructuring (Heimberg & Becker, 2002).

Session 3 to 11 follow the stages of classical psychodrama, which includes warm-up, action, and sharing. Before the warm-up stage, the therapists review homework in order to identify automatic thoughts and thinking errors and use Socratic questioning to help patients with finding a more rational response. The warm-up stage facilitates a safe, supportive and creative atmosphere at the beginning of every session by doing warm-up techniques to prepare patients for action. During the warm-up stage, the therapists ask patients to do a verbal or non-verbal warm-up practice (Weiner & Sacks, 1969). For example, patients are encouraged to get up, move around and select someone to meet as if they have never met them before, but to meet them without using words. After this warm-up stage, the individual who will act as the protagonist is identified (see Table 1 for a description of typical psychodrama roles).

**Table 1:** Description of Typical Psychodrama Roles

Roles	Description
Protagonist	The main character, the session is focused on his/her problem.
Auxiliary Ego	An auxiliary ego is a person that has an important role in the situation chosen by the protagonist in the group and is played by a group member.
Audience	Other patients who observe the action are called audience.
Stage	A semi-circle of chairs is put in the room to create a stage so that the protagonist can act in front of the patients.

Each patient is protagonist at least once during the treatment. The therapist can ask who is ready to work as a volunteer. Alternatively, the therapists can select a protagonist based on what they observed during the preparation in warm-up stage (e.g., sometimes patients express their performance anxiety in the warm-up stage verbally or non-verbally which is appropriate for the selection of the protagonist) or based on information revealed during sharing phase of the previous session (Kumar & Treadwell, 1986).

In the action stage of the therapy sessions, the therapists create a scene with the protagonist, in which an anxiety-provoking situation is acted out. Although role-playing can be an element of CBT, the most important difference between psychodrama and CBT is the aim of role-playing and the manner in which it is executed. In CBGT, role-playing focuses on the thinking process and is used as exposure to change irrational thoughts. In psychodrama, role-playing focuses on emotional expression and it is used to evoke and release emotions (Fisher, 2007). The role-playing can involve past as well as future situations but also feared situations that did not actually happen (Karp & Farrall, 2014). The protagonist can select the auxiliary ego (see Table 1) from the group members. During the action stage, therapists can use various psychodrama techniques, as described in Table 2.

**Table 2:** Description of Psychodrama Techniques and Their Goals for Treatment of SAD

Description	Techniques	Goal
Role reversal	Two individuals first roleplay a situation. Next, the protagonist and the antagonist are asked to change the positions and play the other's role.	Experiencing the role of the other person results in Cognitive change. It helps to correct biased beliefs about how one comes across to others.
Double	A patient of the group plays the protagonist’s inner self and gives a voice to the protagonist’s feelings, thoughts or needs, usually by standing behind the protagonist. The protagonist can accept or reject double’s offers.	Identify automatic thoughts and express suppressed thoughts and feelings during role-playing. It helps the protagonist to explore and expose his/her cognitive distortions.
Empty Chair	The protagonist can talk to an imaginary person that is represented by an empty chair.	Express negative as well as positive feelings.
Mirroring	The auxiliary ego plays the role of the protagonist for a short time. The protagonist stands aside and watches an immediate action and see his/her own behavior, body language and interactions with the other as in a mirror.	Observe themselves through the eyes of the audience works as immediate feedback from the audience (Hammond, 2014) to gain a more realistic view from others’ judgment about his/her performance.
Soliloquy	A monologue in which the patients can express their thoughts and feelings to the audience.	Practice expressing their suppressed thoughts and feelings to the audience to relieve negative beliefs about emotional expression and decrease emotional suppression.

However, during this stage therapists use CBT techniques as well. For example, therapists might shortly stop the scene and use cognitive restructuring to provide alternative thoughts so role-playing can be continued with these alternative thoughts. Which psychodrama technique is used depends on the type of anxiety-provoking situation and is chosen by the therapists with the protagonist’s agreement. For example, role reversal is suitable for social interactions (e.g., talking with strangers, dating, and meeting unfamiliar people), and mirroring is suitable for performing in front of others (e.g., public speaking). Double is used to identify automatic thoughts that can be used for cognitive restructuring and is often used in situations in which someone feels observed (e.g., eating or drinking in front of others, writing in public, going to parties, being at the center of attention, and using public toilets). Finally, empty chair and soliloquy are suitable for traumatic situations where it is helpful to express suppressed emotions.

The last part of each session is sharing or closure. This is a time for patients to discuss the effects the action of the scene had on them and share their feelings and thoughts with the group. The therapists use cognitive restructuring techniques after the action stage to identify automatic thoughts and help patients to correct thinking errors that occurred during role-playing. At the end of each session, the therapists ask patients to provide feedback on therapy session. They also assign exposure in vivo as homework for the protagonist. The other participants not receive homework.

The twelfth and last session is again based on Heimberg and Becker's (2002) protocol and is divided into two parts. The first half is used for practicing with additional exposure, role-playing, and cognitive restructuring. In the second half, the therapists and patients review their development during treatment. That is, they discuss situations that may still be problematic and suggest rational responses can be beneficial in these situations. Finally, therapists help patients to set goals for situations after the end of the formal treatment (Heimberg & Becker, 2002).

#### Statistical Analysis

In total, there were 10 missing values in the BFNE score that were completed each session (6.5 percent). We used a linear mixed model to handle these missing values, which allowed us to still examine if there was an effect of time on the session-by-session BFNE scores. The fixed part included an intercept and a linear effect of time (the pretest BFNE and the scores after completing each treatment session coded as 0, 1, 2, …, 12), the repeated part an autoregressive ARMA11 covariance structure. The effect size of the fixed time effect was expressed as *r* (*r* = *t*/√(*t*^2^ + *df*)). We also estimated the effect size of the pre-post change in terms of Cohen’s *d* which is pre-post change calculated on the basis of the estimated effects of the linear mixed model, divided by the pretest standard deviation (Morris, 2008). The pretest and posttest scores of the other outcomes were compared with paired sample *t*-tests (see De Winter, 2013, for the validity of the *t*-test with small samples). Pre-post effect sizes were calculated in terms of Cohen’s *d* = mean pre-post change divided by pretest standard deviation (Morris, 2008), and Hedges’ *g* (see Table 4 note for the formula). Hedges’ *g* is smaller than conventional Cohen’s *d* but has less bias.

## Results

### Primary Outcomes

A linear mixed model analysis showed that the intervention resulted in a significant reduction of fear of negative evaluation, see Table 3. The pre-post effect size estimated from the linear mixed model on the BFNE was Cohen’s *d* = 1.16.

**Table 3:** Linear Mixed Model Estimates [and 95% Confidence Interval] of Fixed Effects With BFNE as Dependent Variable

Parameter	*b*	*SE*	*df*	*t* (*n*)	*p*	95% CI		Effect Size		
						*LL*	*UL*	*r*	Cohen’s *d* (BL)	Cohen’s *d* (ML)
Intercept	37.64	2.46	6.64	15.28	< .001	31.75	43.53			
Time	-0.68	0.22	11.94	-3.16	.008	-1.15	-0.21	.67	1.16	1.32

*Note.* CI = confidence interval; *LL* = lower limit; *UL* = upper limit; effect size for the fixed effect *r* = *t*/√(*t*^2^ + *df*). Cohen’s *d* (BL) = |b (time)* 12/SD baseline|. Cohen’s *d* (ML) = |standardized beta (Time) * (standardized Time at pretest – standardized Time at posttest)| (Lorah, 2018).

**Figure 1:**
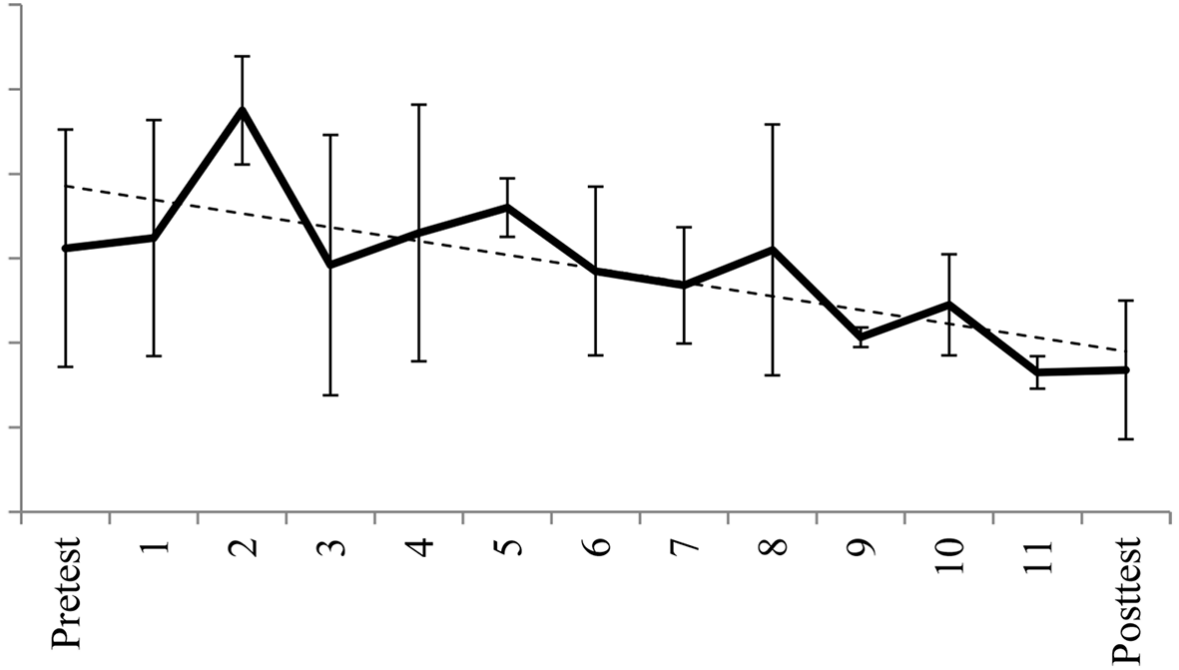
Observed and Estimated (by the Linear Mixed Model) Mean and Standard Deviation (*SD*) of BFNE Every Session by Assessment *Note.* There was a significant linear decrease over time in BFNE scores.

**Figure 2:**
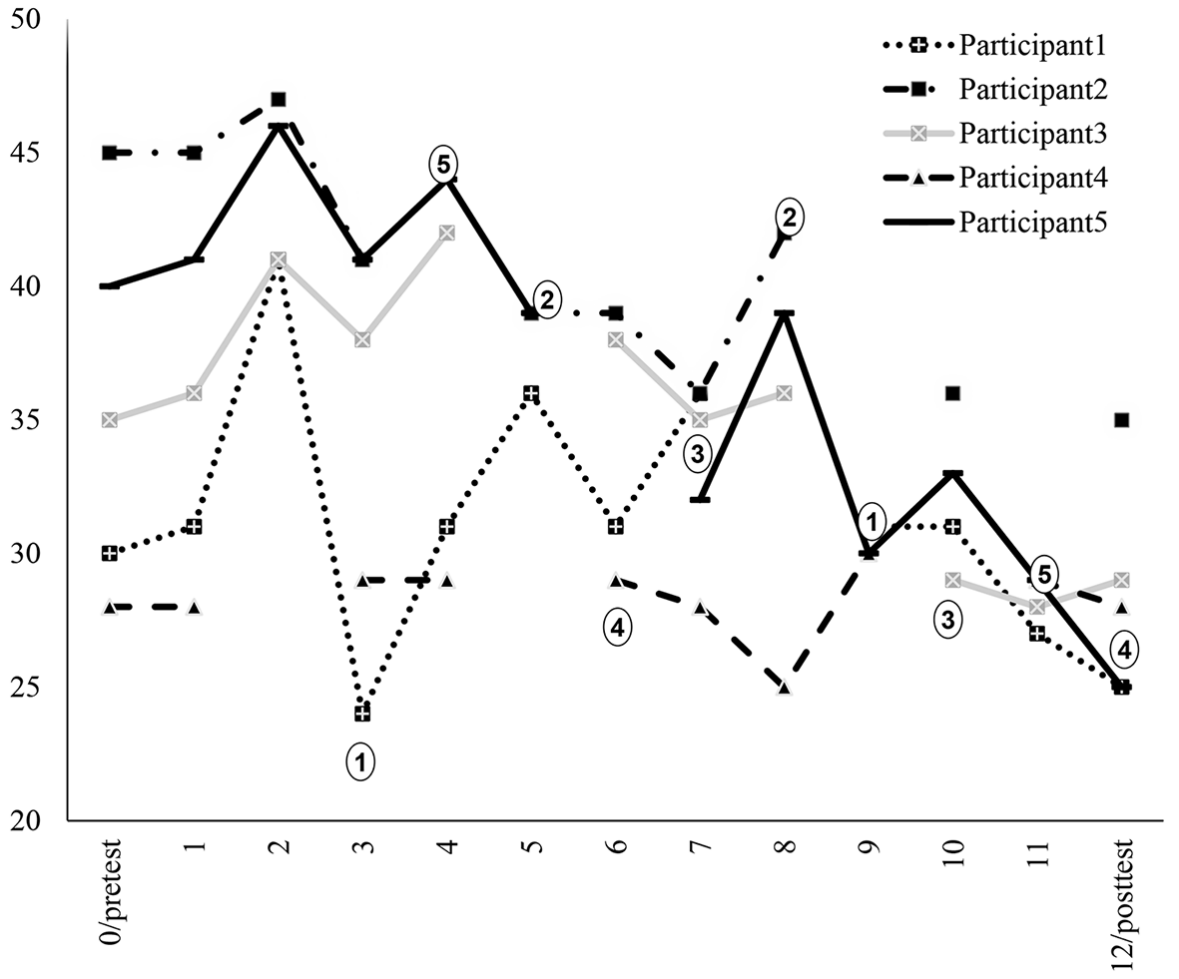
Individual BFNE Scores Over the Period of Treatment *Note.* Dots show who is a protagonist in the session.

Figure 1 illustrates that although the mean score of the BFNE increased after the second session, it then decreased till the end of the treatment. Figure 2 shows the individual BFNE scores per assessment and indicates that in 4 of the 5 participants there was a reduction in BFNE scores. The dots in the figure show at which session each participant had a protagonist role. In 7 of the 10 instances, there was an immediate reduction in BFNE scores after the session.

There was also a significant decrease in social anxiety symptoms assessed with the LSAS (see Table 4).

**Table 4:** Pretest and Posttest Comparison for the CBPT Intervention

Scale	Pre		Post		*t* (4)	*M* difference [CI 99%]			Cohen’s *d*	Hedges*’ g*
	*M*	*SD*	*M*	*SD*		*LL*	*UL*	*p*		
BFNE	35.60	7.02	28.40	4.10	2.86	-4.39	18.79	.046	1.03	0.82
LSAS	99.40	16.99	58.40	24.81	3.82	-8.44	90.44	.19	2.41	1.93
SADS	14.40	5.64	11.80	7.73	1.31	-6.56	11.76	.261	0.46	0.37
PAS	133.20	13.88	131.60	17.21	0.20	-34.67	37.87	.849	-0.12	-0.09
OPQ	56.20	23.18	35.80	16.63	3.22	-8.74	49.54	.032	0.88	0.70
OCQ	64.80	23.47	47.00	26.67	5.95	4.03	31.57	.004	0.76	0.61
BDI	19.60	5.86	12.60	8.20	2.03	-8.82	22.82	.111	1.19	0.96
QOLI	29.40	21.31	36.00	25.17	-0.88	-41.13	27.93	.429	0.31	0.25

*Note*. Observed Means *(M)* and Standard Deviations *(SD)* for the Pre and Post assessment points; results of *t*-test analyses *(t, p-value)* and effect sizes Cohen’s *d* and Hedges’ *g*. BFNE = Brief Fear of Negative Evaluation; LSAS = Liebowitz Social Anxiety Scale; SADS = Social Avoidance and Distress Scale; PAS = Personal Attitude Scale-II; OPQ = Social cost and probability by the Outcome Probability Questionnaire; OCQ = Outcome Cost Questionnaire; BDI = Beck Depression Inventory; QOLI = Quality of Life Inventory. Cohen’s *d* was estimated as *d* = (mean pre-post change)/(pretest *SD*). Hedges’ *g* was calculated as follows: *g* = J*d, with *d* = Cohen’s *d*; J = (1 – 3/(4*df-1)); *df* = *N*-1. The sign of the effect size was chosen so that a positive effect size indicates improvement and negative effect size represents worsening.

### Secondary Outcomes

There was a significant decrease in outcome probability and outcome cost questionnaires. However, there was no significant difference in social avoidance, spontaneity, depression, and quality of life after completing treatment. The test statistics, as well as the effect sizes, are presented in Table 4.

### Reliable Change and Clinical Significant Change

To estimate the rates of clinical significant improvement, we computed the reliable change, clinical significant change, and cutoffs as suggested by Jacobson and Truax (1991) on the primary outcome measures. Moreover, because our sample is too small, we used standard error and test-retest values of two Iranian studies with large samples for BFNE (*SE* BFNE 4.49 from Tavoli, Melyani, Bakhtiari, Ghaedi, & Montazeri, 2009), and LSAS^1^1This was self-report LSAS.
(*SE* LSAS 11.07 from Atrifard et al., 2012). Reliable change (RC) was calculated as difference between post and pretest divided by standard error of change. An RC rate greater than 1.96, is considered as improvement (Jacobson & Truax, 1991; see Table 5). Clinically significant change (CSC) consists of reliable change and a posttest score that falls within mean ± two standard deviations of non-anxious sample, which was 39.86 ± 2*18.98 for LSAS, and 28.7 ± 2*5.9 for BFNE, again using data from two larger studies (Atrifard et al., 2012; Tavoli et al., 2009). As can be seen in Table 5, three of the five patients with LSAS and two of the five patients with BFNE have a clinical significant change after the treatment.

**Table 5:** Within-Participant Changes for the CBPT Intervention on the Primary Outcomes

Participants	BFNE					LSAS				
	Pre	Post	change	RC	below c = 30.97	Pre	Post	change	RC	below c = 57.57
1	30	25	5	N	Y	80	52	28	Y	Y
2	45	35	10	Y	N	104	86	18	Y	N
3	35	29	6	N	Y	83	34	49	Y	Y
4	28	28	0	N	Y	116	37	79	Y	Y
5	40	25	15	Y	Y	114	83	31	Y	N

*Note*. RC = Reliable Change; BFNE = Brief Fear of Negative Evaluation; LSAS = Liebowitz Social Anxiety Scale (LSAS); Y = yes; N = no.

### Feedback From Patients

In the course of the treatment, role reversal and double were the most frequently used techniques in CBPT based on therapists’ post-session reports. After sessions, patients reported that role reversal was a helpful technique that enables them to expose themselves to anxiety-provoking social situations. They further reported that cognitive restructuring as it was integrated into techniques in the action stage, helped them to understand CBT concepts in a more experiential way. Patients also experienced some warm-up techniques (e.g., forming a band by playing their invisible musical instruments) as anxiety-provoking and embarrassing situations, but they finally evaluated them as helpful warm-up techniques to decrease anxiety.

## Discussion

CBPT balances a focus on cognition and behavior through CBT techniques, and emotion during psychodrama techniques in action. The results from this pilot study supported that integrating CBGT and psychodrama might be considered as a new treatment for patients diagnosed with SAD. Also, the fact that patients continued the treatment until the last session indicates that CBPT was acceptable for patients.

The pilot indicated that the treatment was effective in the core area of SAD. Social anxiety, as assessed by the LSAS, reduced significantly from pre to posttest. The current study showed a high effect size on the LSAS (pre-post effect size Hedges’ *g* = 1.93) in comparison to the pre-post effect sizes of other studies using Heimberg’s CBGT on the LSAS (Blanco et al., 2010, *g* = 0.56; Bjornsson et al., 2011, *g* = 0.61; Hayes-Skelton and Lee, 2018, *g* = 0.82; Hedman et al., 2011, *g* = 0.99; Heimberg et al., 1998, *g* = 0.75).

Significant improvements were also found on the two cognitive measures of cost and probability estimates of negative outcomes. This suggests that CBPT can change cognitive processing biases to decrease social anxiety in SAD. Our findings are in line with research that reported changes in probability or cost estimates after CBT, which in turn related to therapeutic changes in social anxiety symptoms (Foa et al., 1996; Gregory et al., 2015; Hofmann, 2004; Lucock & Salkovskis, 1988). Hamamci (2002) also showed that integrating CBT and psychodrama techniques leads to a reduction in cognitive distortions related to interpersonal relationships. It is conceivable that the use of psychodrama techniques contributed to a decrease in estimated social cost and probability because it helped patients to experience a disconfirmation of their expectations. However, because in the current study CBT and psychodrama techniques were integrated, it is not clear how much change results from psychodrama techniques alone. Future research should reveal if that CBPT is more effective in decreasing negative beliefs than CBT or psychodrama alone.

Likewise, fear of negative evaluation also reduced during treatment with a pre-post effect size of Hedges’ *g* = 0.82 on BFNE scores, which is in line with the pre-post effect sizes of studies using CBGT in the treatment of SAD (Bjornsson et al., 2011; Heimberg et al., 1998).

The decline of fear of negative evaluation was not consistent in the course of treatment. After the second session, there was an increase in fear. This might be due to the announcement in the second session of the start of in-session exposure and role-playing in the third session. However, the increase was only temporary, and social anxiety decreased significantly till the end of treatment. Fear of negative evaluation decreased immediately after 7 of the 10 sessions in which a patient was the protagonist, showing an overall immediate positive effect of being protagonist on social anxiety symptoms in a small sample. Why being a protagonist was not always followed by a decrease in BFNE is not clear. This might be due to the patients’ attitude toward role-playing or the level of expression of emotions, or other factors. Clearly, further work in large clinical trials is required to gain a better understanding of the effects of being the protagonist in social anxious patients.

Next to social anxiety outcomes there were several other outcomes measures. These showed that there were no significant differences between pre and posttest in avoidance, spontaneity, depression symptoms and quality of life. The lack of significant effects on the measure of spontaneity is rather surprising, given the prominent position spontaneity has in the theory of psychodrama. Perhaps the spontaneity measure that we used is not sensitive to change because the items that were used describe spontaneity more as a stable personality trait than a characteristic that can easily be changed during a short CBPT treatment. However, Moreno (1953) noted that especially spontaneity can be enhanced during psychodrama and that it is an important mechanism of clinical change (Moreno, 1953). Further research is required to examine if the current lack of change in spontaneity is due to the type of measure or if the short integrated CBPT is not suitable to change spontaneity. The lack of significant effects on avoidance, depression, and quality of life might relate to the limited power of this pilot study, as the changes are in the direction of improvement, and are in the range of effect sizes of previous studies, or exceed them. That is, the finding on avoidance, depression, and quality of life are consistent with previous studies: Avoidance with a pre-post effect size of Hedges’ *g* = 0.37 on SADS scores, while Heimberg’s studies using CBGT in the treatment of SAD resulted in a pre-post SADS effect size of Hedges’ *g* = 0.29 (Heimberg et al., 1990), and Hedges’ *g* = 0.17 (Heimberg et al., 1998); Depression with a pre-post effect size of Hedges’ *g* = 0.96 on BDI scores, which is in line with previous studies using CBGT in the treatment of SAD that found pre-post BDI effect sizes of Hedges’ *g* = 0.78 (Heimberg et al., 1990), and Hedges’ *g* = 0.82 (Koszycki, Benger, Shlik, & Bradwejn, 2007); Quality of life with a pre-post effect size of Hedges’ *g* = 0.25 on QOLI scores, which is in line with other studies using CBGT in the treatment of SAD finding small pre-post QOLI effect sizes of Hedges’ *g* = 0.28 (Hayes-Skelton & Lee, 2018), and Hedges’ *g* = 0.44 (Koszycki et al., 2007).

An important limitation of the present study is that our sample size was small (5 patients) limiting the external validity of the results. Besides, this was an uncontrolled study and the internal validity study is limited by the lack of a control group. Moreover, the LSAS assessors were not blind to the timing of the interviews (before or after treatment). There was no follow-up assessment into also, thus it is unclear whether the results were maintained or whether there were further changes. This is in particular important for outcomes like avoidance, depression, and quality of life that might show a delayed response to treatment. Furthermore, integrating psychodrama and CBT in therapeutic practice usually includes 16 sessions (Treadwell, Dartnell, Travaglini, Staats, & Devinney, 2016). However, the current CBPT protocol consists of twelve sessions to make it comparable to CBGT in future random clinical trials. Nevertheless, the effects of CBPT might be larger with 16 sessions. Future studies might investigate different lengths of treatment. The results of this pilot are promising, but it is necessary to do research in a randomized controlled trial with follow-up assessments to compare this treatment to CBGT alone and psychodrama alone.
